# Transcriptome analysis of fowl adenovirus serotype 4 infection in chickens

**DOI:** 10.1007/s11262-019-01676-w

**Published:** 2019-07-01

**Authors:** Guangcai Ren, Han Wang, Miaorong Huang, Yuanyuan Yan, Fan Liu, Ruiai Chen

**Affiliations:** 1 Key Laboratory of Biotechnology and Drug Manufacture for Animal Epidemic Prevention, Ministry of Agriculture, Zhaoqing, China; 2 College of Veterinary Medicine, South China Agriculture University, Guangzhou, China; 3 Zhaoqing Institute of Biotechnology Co., Ltd., Zhaoqing, China

**Keywords:** Fowl adenovirus serotype 4, Hydropericardium–hepatitis syndrome, RNA-seq, Molecular pathogenesis, Differentially expressed genes

## Abstract

Fowl adenovirus serotype 4 (FAdV-4) is a causative agent of inclusion body hepatitis and hydropericardium–hepatitis syndrome. These diseases cause considerable economic losses in the global poultry industry and are significant stressors for infected chickens. However, the molecular mechanisms of FAdV-4 pathogenesis are poorly understood. In the present study, we identified differentially expressed genes from the livers of FAdV-4-infected chickens using RNA-seq at 7, 14 and 21 days after FAdV-4 infection. We identified 2395 differentially expressed genes at the three time points. These genes were enriched in variety of biological processes and pathways including PPAR and Notch signaling, cytokine–cytokine receptor interactions and Toll-like receptor signaling pathways. The transcriptional data were validated by quantitative real-time PCR. Our results will assist in the understanding of the molecular pathogenesis of FAdV-4 infection and for developing novel antiviral therapies.

**Electronic supplementary material:**

The online version of this article (10.1007/s11262-019-01676-w) contains supplementary material, which is available to authorized users.

## Introduction

The fowl adenoviruses (FAdV) are all members of the genus *Aviadenovirus* and are classified as five species (FAdV-A to FAdV-E) comprised of 12 serotypes (FAdV-1 to 8a and -8b to 11) based on restriction enzyme digest patterns and serum cross-neutralization [1]. The FAdV are globally distributed with the first clinical cases appearing in Pakistan [2] and subsequently in the USA [3], Germany [4], Canada [5], India [6], China [7], Korea [8], Japan [9], Mexico [10] and Poland [11].

FAdVs cause huge economic losses to the poultry industry from outbreaks of gizzard erosion [12], hepatitis–hydropericardium syndrome (HHS) [13], respiratory disease [14] and inclusion body hepatitis (IBH) [15]. IBH can be caused by all 12 serotypes of FAdVs and is characterized by a congested and enlarged liver with necrosis and petechial hemorrhaging [16]. HHS is a severe clinical condition caused by FAdV-4 resulting in accumulation of fluid in the pericardial sac with nearly 100% mortality [10].

FAdV-4 plays a primary role in the etiology of IBH/HHS although its molecular pathogenesis has been only recently investigated. The response of leghorn male hepatocellular (LMH) cells to FAdV-4 infection has implicated the Toll-like receptor (TLR) and MAPK signaling pathways [17]. Moreover, the non-pathogenic strain ON1 induced liver expression of interferon (IFN)-γ and interleukin (IL)-10 [18].

A primary target organ of FAdV-8b is the liver and hepatic lesions are correlated with three stages of disease progression. These stages are incubation (1–3 dpi), degeneration (4–7 dpi) and convalescence (14 dpi) [19]. Epidemiological investigations of FAdV-4 on 25 different commercial Chinese chicken flocks indicated that mortality peaked after 3–4 days, declined after 9–14 days and was then followed recovered [7]. However, the virus remains latent in the chicken organ for a long period. Several reports showed that the viral strain was still present in liver until day 28 after experimental infection [10, 18, 20]. In general, a low viral load persisted until 21 days post infection (dpi) in the absence of obvious symptoms of HHS [20].

Specific pathogen-free (SPF) chickens infected with FAdV-4 ON1 developed a strong antibody response at 7, 14, 21 and 28 dpi [18]. In other experiments, specific anti-FAdV-4 HLJFAd15 antibody appeared around 7 dpi and continued to rise until 35 dpi [21]. Our previous study using FAdV-4 strain GX-1 showed that a similar antibody response pattern [22]. In addition, infection with FAdV-4 caused depletion of B and T cells in lymphoid organs and suppressed the humoral and cell-mediated immune responses [23].

Considering these host response patterns to FAdV-4 infection, we used in vivo infection model SPF chickens to analyze the host transcriptome at 7, 14, and 21 dpi using RNA-seq to identify how the hosts respond to FAdV-4 infection.

## Materials and methods

### Ethics statement

Our study was approved by the Animal Care and Use Committee of Guangdong Province, China. All animal procedures were performed according to guidelines developed by the China Council on Animal Care and protocol approved by Animal Care and Use Committee of Guangdong Province, China.

### Virus

FAdV-4 strain GX-1 (Genbank Accession No. MH454598) was isolated from a commercial broiler chicken in 2017 in Guangxi Province, China, and was stored and propagated by our laboratory.

### Experimental animals and tissue collection

Fifty 10-day-old SPF White Leghorn chickens were randomly divided into two groups of 25 birds each. One group was inoculated with FAdV-4 GX-1 and the other was inoculated with sterile PBS and used as negative control. Inoculations were given intramuscularly (i.m.) using 200 µL of inoculum containing 10^2^ tissue culture infective doses (TCID_50_) of virus. Challenged chickens that died due to infection were not utilized for RNA-seq library construction. Three chicks from each group were selected randomly for necropsy at 7, 14 and 21 dpi. Our transcriptome samples were taken from six groups that included 7, 14 and 21 dpi from mock and infected animals. Each group was processed with three independent replicates. Liver samples were collected and immediately frozen in liquid nitrogen for viral DNA detection and RNA isolation or fixed in 10% neutralized buffered formalin for histological processing. Livers were processed routinely for haematoxylin and eosin and immunohistochemical staining as previously described [24].

### RNA-seq library construction

Total RNA was extracted from frozen livers using Trizol (Invitrogen Thermo Fisher, Waltham, MA, USA) according to the manufacturer’s instructions and treated with RNase-free DNase I (Takara Bio, Shiga, Japan) to remove potential genomic DNA contamination. RNA quantity and quality was assessed using UV spectroscopy with a Nano Drop 2000 (Thermo Fisher) instrument. RNA integrity was checked using an Agilent Bioanalyzer 2100 (Agilent Technologies, USA). After total RNA was extracted, mRNA was enriched by Oligo (dT) beads. Then the enriched mRNA was fragmented into smaller pieces with fragmentation buffer and reverse-transcripted into cDNA with random primers. Second-strand cDNA were synthesized in the presence of DNA polymerase I, RNase H, dNTP and buffer. Then, the synthesized cDNA fragments were purified with QiaQuick PCR extraction kit (QIAGEN, German), end repaired, poly (A) added, and ligated to Illumina sequencing adapters. The ligation products were size selected by agarose gel electrophoresis. PCR amplified, and sequenced using Illumina HiSeqTM 2500 by Gene Denovo Biotechnology Co. (Guangzhou, China).

### Bioinformatics

High-quality sequencing reads were generated by filtering reads containing adapters, > 10% of unknown nucleotides and those with > 50% of low-quality (*Q* value ≤ 20) bases. Short reads were aligned using Bowtie2 and mapped to rRNA database [25]. The rRNA mapped reads were removed and the remaining reads were aligned with the *Gallus gallus* reference genome using TopHat2 (V. 2.0.3.12) [26].Transcript reconstruction was carried out using Cufflink [27]. Transcripts from replicate samples were grouped and merged into a final comprehensive set of transcripts for further downstream differential expression analysis. Gene abundance was quantified using RSEM software [28]. Gene expression trends from 7 to 21 dpi were analyzed and clustered using the software of Short Time-series Expression Miner (STEM) [29]. DEGs belonging to the same cluster were proposed to have similar expression pattern with each other. The clustered profiles of DEGs with *p* < 0.05 were considered as significantly different from the reference set. Differentially expressed genes (DEG) were identified using edgeR software (https://bioconductor.org/packages/release/bioc/html/edgeR.html). Transcripts with log_2_|fold changes| > 1 and *p* value < 0.05 were cataloged as significant DEGs. Gene Ontology (GO) ((http://www.geneontology.org/) and KEGG pathway analysis (https://www.kegg.jp/) were used to further classify DEGs. GO classification was performed using WEGO software [30]. The RNA-seq raw data have been deposited in the NCBI SRA database (SRA accession: PRJNA498911).

### Real-time PCR

A group of seven DEGs were selected randomly for validation by RT-PCR. These were NFIL3, AKT1, PLP1, TLR2A, FABP2, MBL2 and PIGR. The RNA samples used for the RT-PCR assays were the same as those used for the DEG experiments and were independent RNA extractions from biological replicates. The cDNA was synthesized from 3 μg of total RNA for each sample. Primers (Table S1) were designed using Primer Premier 5.0 software (http://www.premierbiosoft.com/primerdesign/). Reactions were performed with SG Fast qPCR Master Mix (Sangon Biotech, Shanghai, China) in a StepOnePlus Real-Time PCR system (Thermo Fisher) according to the manufacturer's instructions. The reaction parameters were 95 °C for 3 min, followed by 45 cycles of 95 °C for 3 s, 60 °C for 30 s, and 72 °C for 30 s. Each sample was run in triplicate. Relative expression levels were normalized to the endogenous control gene β-actin and expression ratios were calculated using the $2^{{ - \Delta \Delta C_{\text{t}} }}$ method.

### Viral DNA detection of liver samples

PCR primers were designed based on hexon gene of fowl adenovirus C isolate ON1 (GenBank GU188428): 5′-GGA CCT CCA ACA GTT CAT TT-3′ and 5′-AGC CAG CGG GTT GTA AGC-3′. PCR reactions were performed using the following protocol: 95 °C for 5 min, followed by 34 cycles of 95 °C for 30 s, 55 °C for 30 s, and 72 °C for 30 s, followed by a final elongation step of 10 min at72 °C. The product length was 300 bp.

## Results

### Clinical and pathologic features of the FAdV-4-infected chickens

**Fig. 1:**
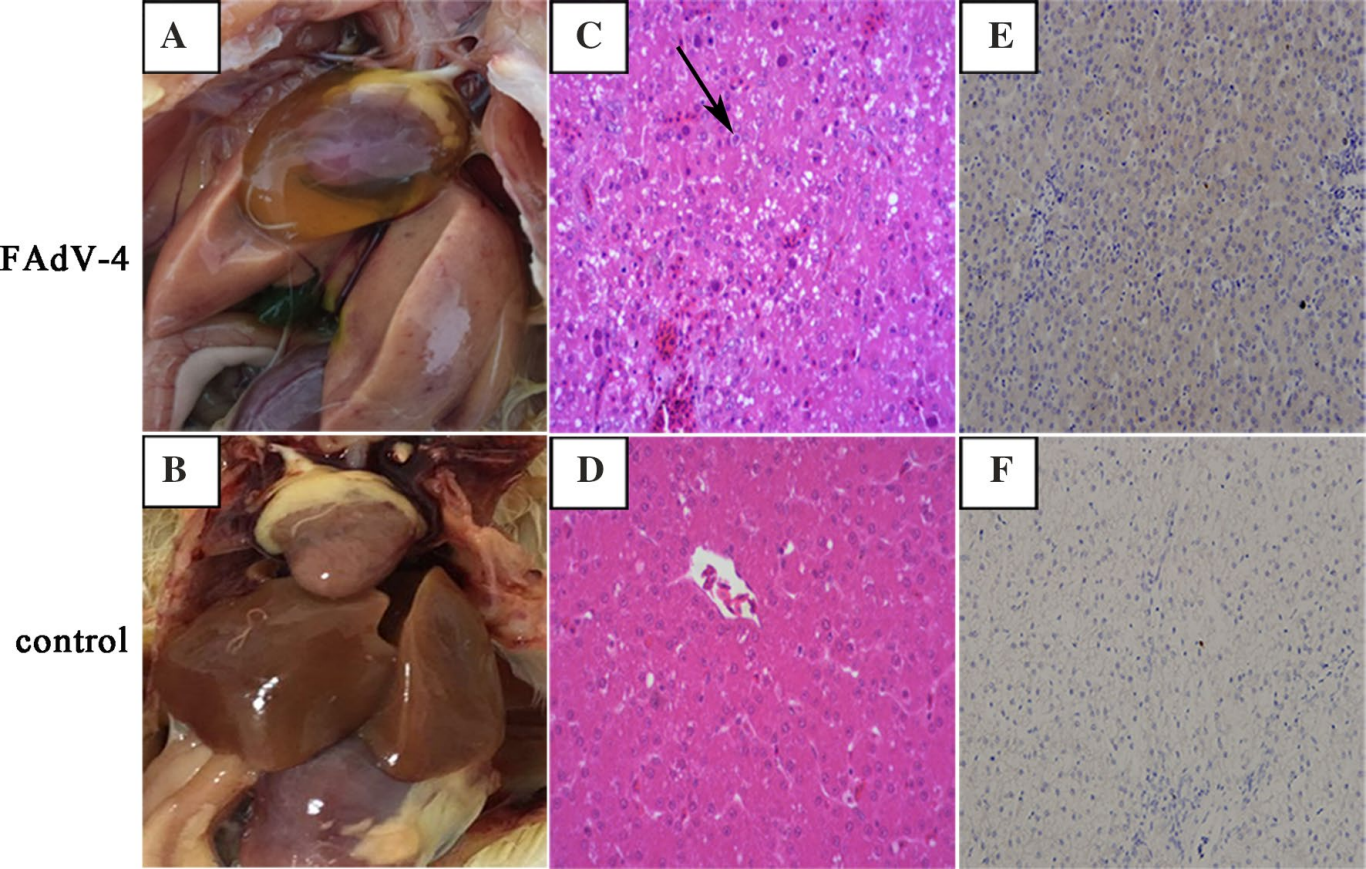
Pathologic examination of livers infected with the FAdV-4 GX-1 strain on day 7 post-infection. **a** Gross lesions in liver. **c** Hematoxylin–eosin staining in liver. Solid arrows indicate viral inclusion bodies **e** immunohistochemical analysis of liver. **b**, **d**, **f** Negative controls

**Fig. 2:**
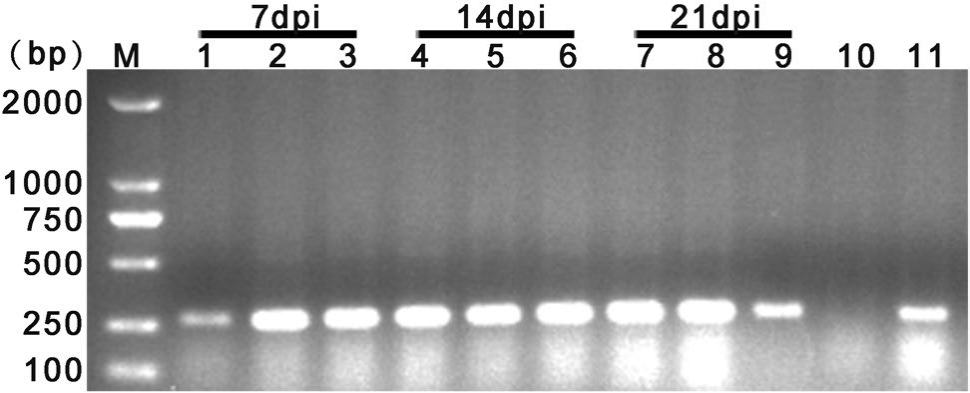
PCR analysis of viral DNA from infected livers. Agarose gel electrophoresis of PCR amplicons visualized by EtBr staining and UV light. Lane M: DL 2000 bp marker, lane 1–3: viruses DNA in the liver sample at 7 dpi, lane 4–6: viruses DNA in the liver sample at 14 dpi, lane 7–9: viruses DNA in the liver sample at 21 dpi, lane 10: negative control, lane 11: positive control

Chickens experimentally infected with FAdV-4 strain GX-1 were unable to move and showed depression, ruffled feathers, trembling, lethargy and loss of appetite within 2–5 days post-infection (dpi). Mock-infected chickens did not show any obvious clinical signs or symptoms. At necropsy, livers from infected chickens were swollen and yellow brown with necrotic foci (Fig. 1a). No significant gross lesions were present in control chicken livers (Fig. 1b). Histological analysis indicated eosinophilic intranuclear inclusion bodies in hepatic cells (Fig. 1c). No lesions were observed in the corresponding tissues of chickens in the control group (Fig. 1d). Immunohistochemistry of FAdV-4-infected chickens indicated that fowl adenovirus was present in liver tissues (Fig. 1e). PCR analysis result showed that viral DNA was detected in liver samples at 7 dpi, 14 dpi and 21 dpi (Fig. 2).

### Transcriptome sequencing

**Fig. 3:**
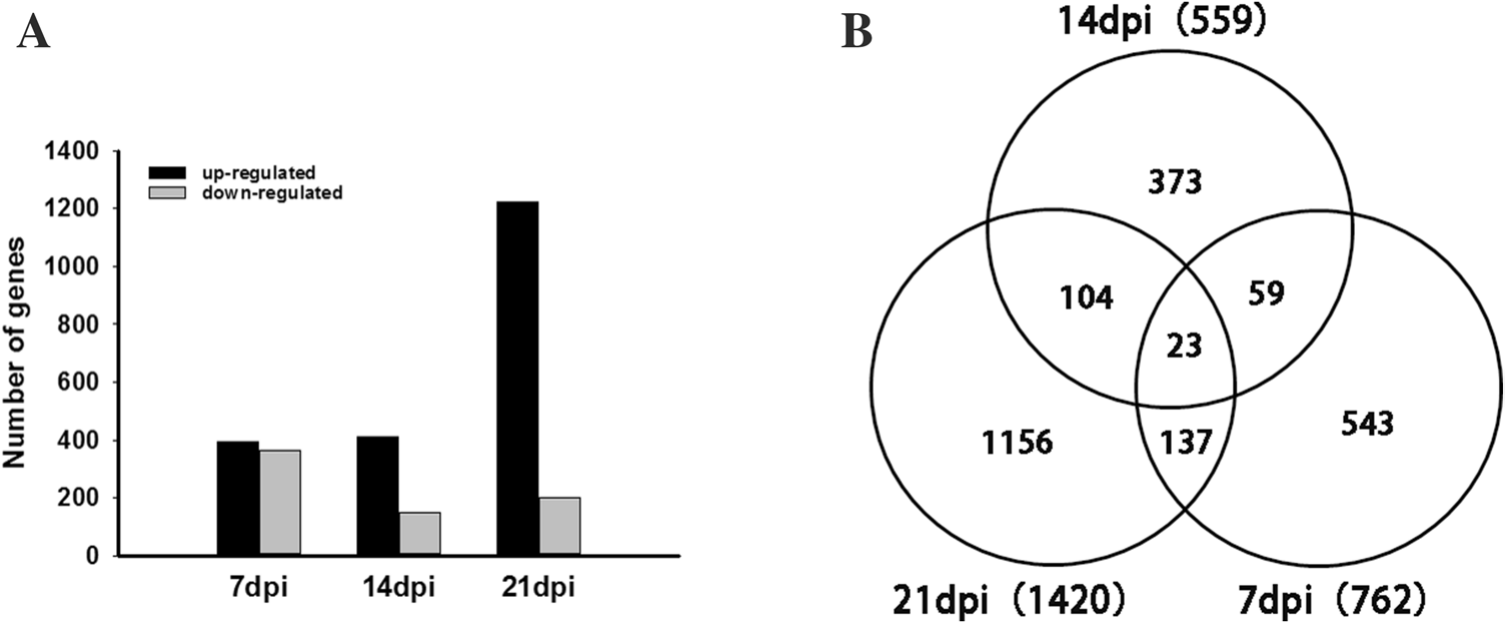
Identification of differentially expressed genes. **a** Numbers of differentially expressed genes. **b** Venn diagram of differentially expressed genes during the course of infection

Approximately, 40–73 million clean reads were sequenced and filtered using RNA-seq technique from 18 cDNA libraries prepared from FAdV-4-infected and non-infected chickens (Table S2). After filtering, the high-quality clean reads were aligned and mapped to rRNA database (Table S3). The rRNA mapped reads will be removed. The unmapped reads were aligned with the chicken genome and all samples had mapping ratios from 85 to 90% (Table S4). Moreover, 18,346 transcripts were observed and were filtered by the thresholds of *p* value < 0.05 and |log_2_ fold-change| > 1. Under these criteria, 2395 DEGs were identified in chickens after FAdV-4 infection at the three time points between by a comparison of the two groups. During the time course of experimental infections we identified 762 DEGs at 7 dpi (396 upregulated and 366 downregulated), 559 DEGs at 14 dpi (410 upregulated and 149 downregulated) and 1420 at 14 dpi (1221 upregulated and 199 downregulated) (Fig. 3a). Overall, 23 genes were differentially expressed at all three time points (Fig. 3b).

**Fig. 4:**
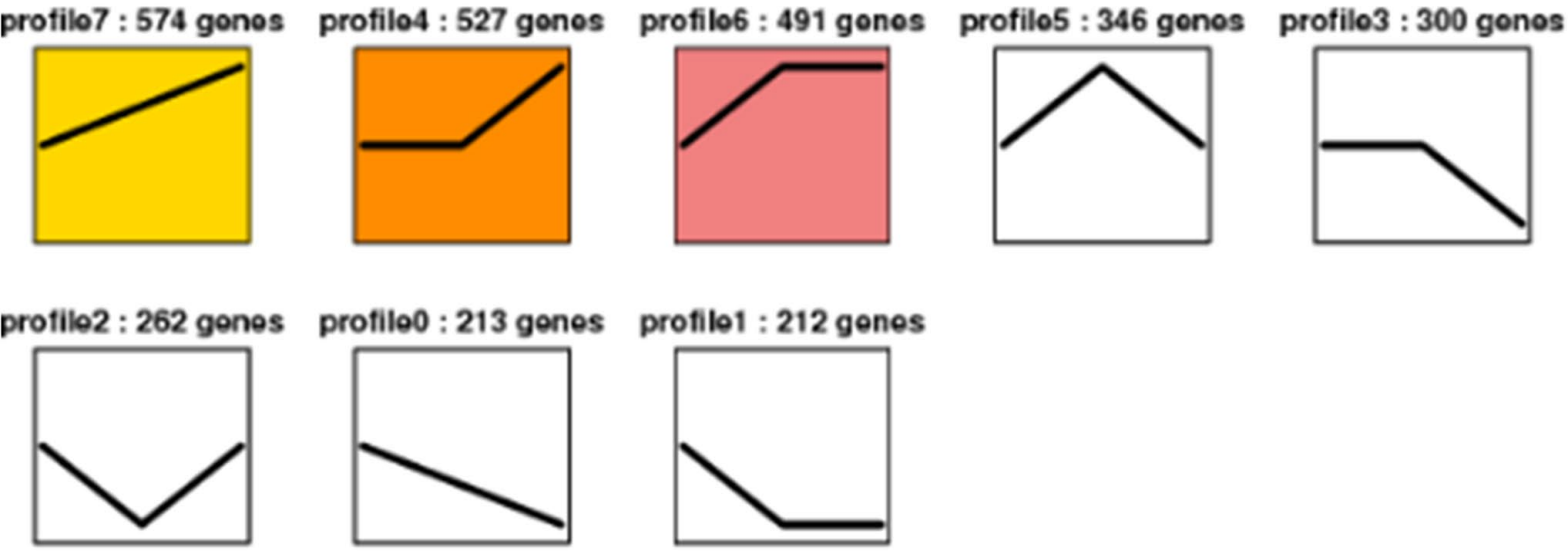
Clusters of differentially expressed genes and numbers of differentially expressed genes in different clusters. Numbers indicated profiles or gene numbers. Significantly different profiles were represented by different background colors

To better understand the dynamic changes of gene expression in liver during all the three time points, further analyses of the DEGs were also performed between the two stages of FAV-7d-vs-FAV-14d, FAV-7d-vs-FAV-21d, FAV-14d-vs-FAV-21d, respectively. Finally, a total of 2925 genes were identified as DEGs among the three stages. The DEGs were classified into eight clusters according to their expression patterns throughout the process of infection of FAdV-4 (Fig. 4). Three significant expression profiles (profile 4, profile 6, and profile 7) were identified. As shown in Fig. 4, significantly different profiles were represented by different background colors. The 527 DEGs in profile 4 were significantly present no change between 7 to 14 dpi and upregulated from 14 to 21 dpi. Profile 6 contained 491 DEGs in a pattern reverse of that in profile 4 rend. Profile 7 included 574 DEGs that were upregulated from 7 to 21 dpi. Among the 8 gene expression profiles, profile 0, profile 1 and profile 2, which indicate a similar expression level between 7 and 14 dpi and a different expression level between 14 and 21 dpi, contained 213, 212 and 262 DEGs, respectively. Profile 3 and profile 5 displayed patterns of downregulation between 14 and 21 dpi and a different expression level between 7 and 14 dpi contained 300 and 316 DEGs.

### DEGs were enriched in various biological processes and pathways

**Fig. 5:**
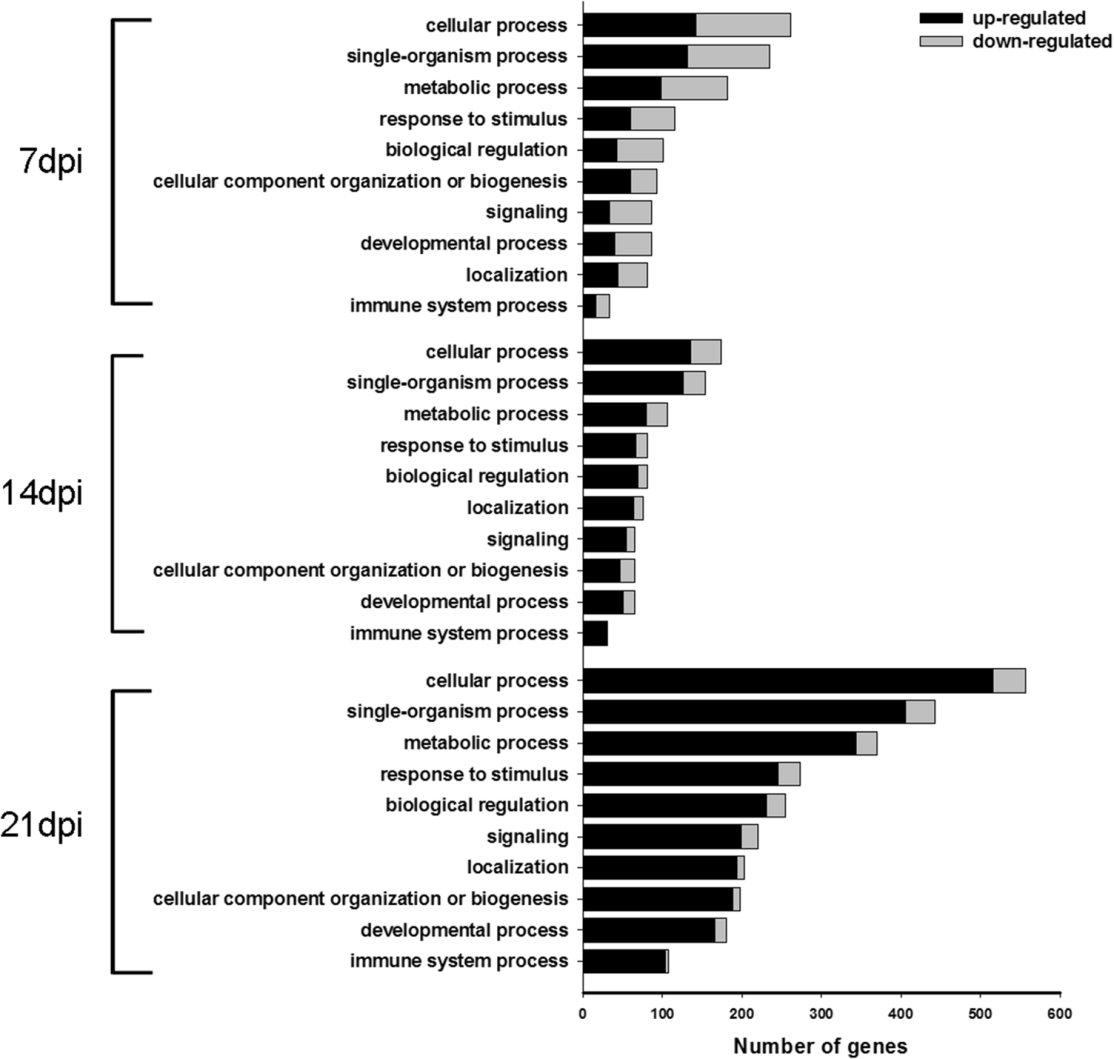
Top 10 GO categories significantly enriched in biological process at 7, 14, and 21 dpi

The DEGs were then functionally classified using GO and KEGG analysis. According to the GO functions, the annotated DEGs were classified into biological processes, cellular components, and molecular functions. Top ten biological processes were significantly (*p* < 0.05) enriched by GO analyses of the up- and downregulated genes at the three time points (Fig. 5). The GO terms included cellular process, single-organism process, metabolic process, and response to stimulus, biological regulation, cellular component organization or biogenesis, signaling, developmental process, localization and immune system process. These were all significantly enriched at all three time points (*p* < 0.05). However, the number of genes between time points differed.

**Fig. 6:**
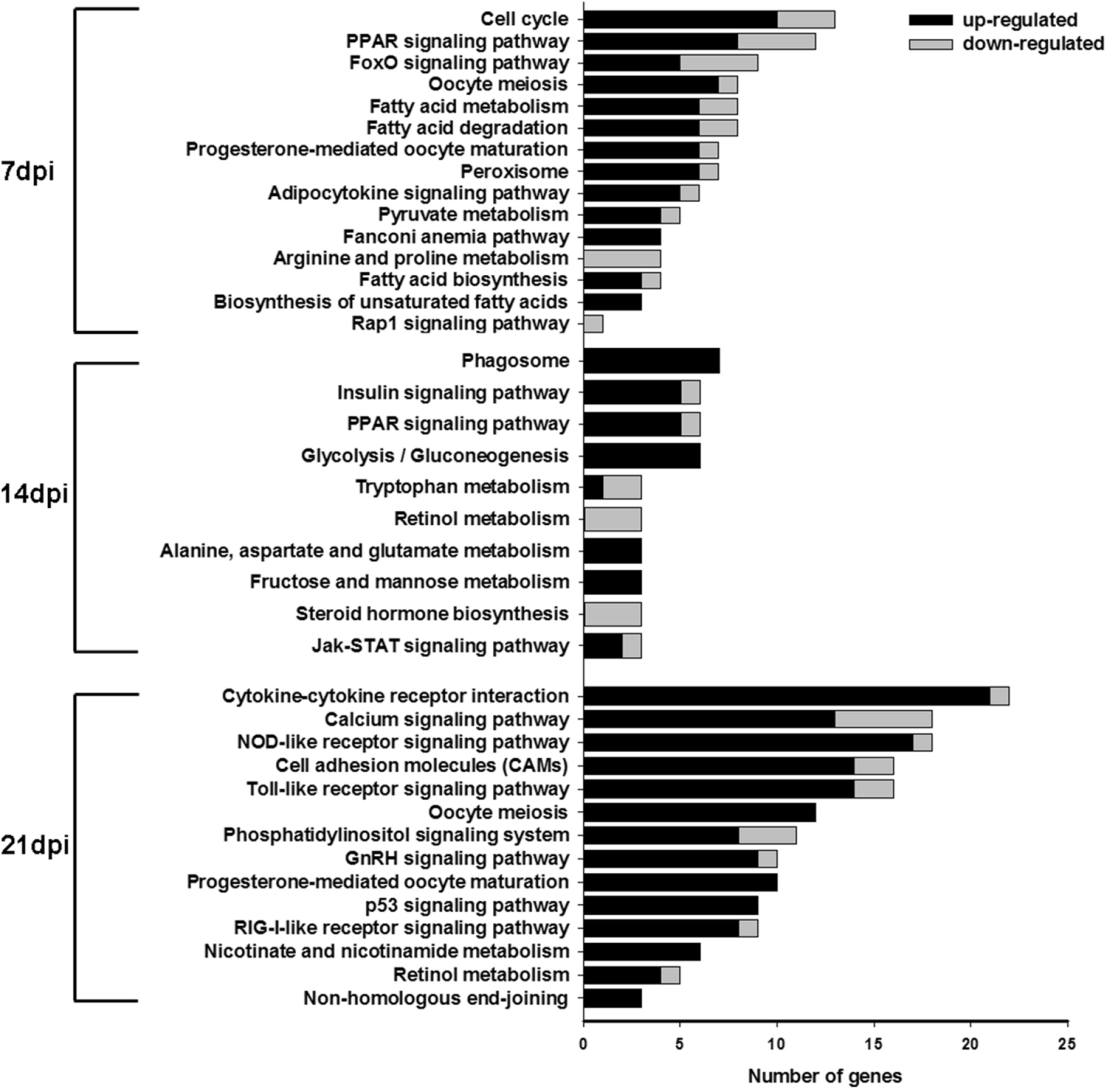
KEGG pathways significantly enriched in biological process at 7, 14, and 21 dpi

To characterize the functional consequences of gene expression changes associated with infection with FAdV-4, we performed pathway analysis based on the KEGG database. We identified 39 KEGG pathways that were significantly enriched at the three time points (Fig. 6). Among the 39 KEGG pathways, there were 15 at 7 dpi, 10 at 14 dpi, and 14 at 21 dpi. In addition, there were several immune system-related signaling pathways that were enriched at every time point. Overall, the numbers of upregulated genes were greater than downregulated genes at the different time points.

### Lipid metabolism and immune signal pathways were activated after FAdV-4 infection

Fourteen pathways related to innate immunity and inflammation were affected at the three time points. Both the host innate immune response and host lipid metabolism were changed during the viral replication and proliferation stages. For instance, in the cytokine–cytokine receptor interaction pathway, TNFSF15, KIT, IL22RA2, CSF3R, CCL19, CCL20, IFN-A, IL18, PDGFRA, INHBB, CX3CL1, OSMR, IL6ST, IFNG, LIFR, CCR6, IL1R1, FAS, IL22RA1, KIT, IL21R, CSF2RA, CCR5 were upregulated, whereas IL18, TGFB3, TNFRSF13B were downregulated. In the Toll-like receptor signaling pathway, TLR1, TLR2, STAT1, TLR7, IFN-A, NFKBIA, AKT1, CD86, TLR4, PI3 K were upregulated at 21 dpi, while IFN-A, FOS, JUN were downregulated at 7 and 14 dpi. In the Notch signaling pathway, RBPJL, EP300, DTX3L, CREBBP were upregulated, and HES1, CREBBP were downregulated. The IFN-A gene was enriched in several signaling pathways including the cytokine–cytokine receptor interaction pathway, the Toll-like receptor signaling pathway, the NOD-like receptor signaling pathway and the RIG-I-like receptor signaling pathway (Table 1).

**Table 1:** KEGG pathways related to lipid metabolism signal pathway and immune response at three time points

Pathway ID	KEGG pathway	7 dpi		14 dpi		21 dpi	
		Upregulated genes	Downregulated genes	Upregulated genes	Downregulated genes	Upregulated genes	Downregulated genes
ko03320	PPAR signaling pathway	PCK1, ACAA1, ACOX1,EHHADH, CPT1A, ACSL1, FABP1, FABP2	ACSBG2, MMP-1, GK	FABP3, PCK1, FABP1, FABP2, MMP1	ANGPTL4	FABP3, PLIN2, PPARG, SCD, ANGPTL4	GK
ko04010	MAPK signaling pathway	STMN1, RPS6KA5	FLNA, FGF1, TGFB3, DUSP4, FOS, JUN, HSPA2	MAP3K8	HSPA2	PRKACB, DUSP8, PDGFRA, FGF23, RAP1A, FAS, RASA2, AKT1, RPS6KA2, IL1R1, DUSP4, RPS6KA5, MAP3K1, PLA2G4A	CACNA1D, FOS
ko04060	Cytokine–cytokine receptor interaction	TNFSF15, KIT	IFN-A, IL18, TGFB3, TNFRSF13B	IL22RA2, IFN-A, CSF3R		CCL19, CCL20, IFN-A, IL18, PDGFRA, INHBB, CX3CL1, OSMR, IL6ST, IFNG, LIFR, CCR6, IL1R1, FAS, IL22RA1, KIT, IL21R, CSF2RA, CCR5	IFN-A
ko04068	FoxO signaling pathway	PLK4, PCK1, PLK1, CCNB2	TGFB3, CDKN1A, INS, p15	PCK1,CDKN1A, G6PC2		CCND2, PI3 K, PLK4, PTEN, AKT1, CDKN2B, CCNG2, FBXO25, PLK3, PRKAA2, CDKN1A, G6PC2	
ko04115	p53 signaling pathway	CDK1, RRM2, GTSE1, CCNB2	CDKN1A	CDKN1A		CCND2, PTEN, FAS, APAF1, ATR, CCNG2, GTSE1, CDKN1A, SESN1	
ko04310	Wnt signaling pathway		WNT6, JUN, Wnt10a		WNT11	CCND2, Chd8, PRKACB, NFATC2, MMP7	
ko04330	Notch signaling pathway	RBPJL, EP300			HES1, CREBBP	DTX3L, CREBBP	
ko04350	TGF-beta signaling pathway		TGFB3, CDKN2B	PPP2R1A		INHBB, IFNG, CDKN2B	
ko04620	Toll-like receptor signaling pathway		IFN-A, FOS, JUN	IFN-A, MAP3K8		TLR1, TLR2, STAT1, TLR7, IFN-A, NFKBIA, AKT1, CD86, TLR4, PI3 K	IFN-A, FOS
ko04621	NOD-like receptor signaling pathway	CATHL2	IFN-A, IL18, JUN	IFN-A		STAT1, IFN-A, IL18, ATG5, PRKCD, CARD9, NFKBIA, ITPR3, P2RX7, CYBB, NAMPT, IFN-A, TLR4	IFN-A
ko04622	RIG-I-like receptor signaling pathway		IFN-A	IFN-A		IFN-A, ATG5, NFKBIA, MAP3K1, TRIM25, DHX58	IFN-A
ko04623	Cytosolic DNA-sensing pathway		IFN-A, IL18	IFN-A		IFN-A, IL18, NFKBIA	IFN-A
ko04630	Jak-STAT signaling pathway		PTPN2	IL22RA2, PTPN2	PTPN2	CCND2, PTPN2	
ko04672	Intestinal immune network for IgA production	PIGR	TNFRSF13B	BLB1		LICOS, ICOS, AICDA, CD86	

### Validation of RNA-seq results

**Fig. 7:**
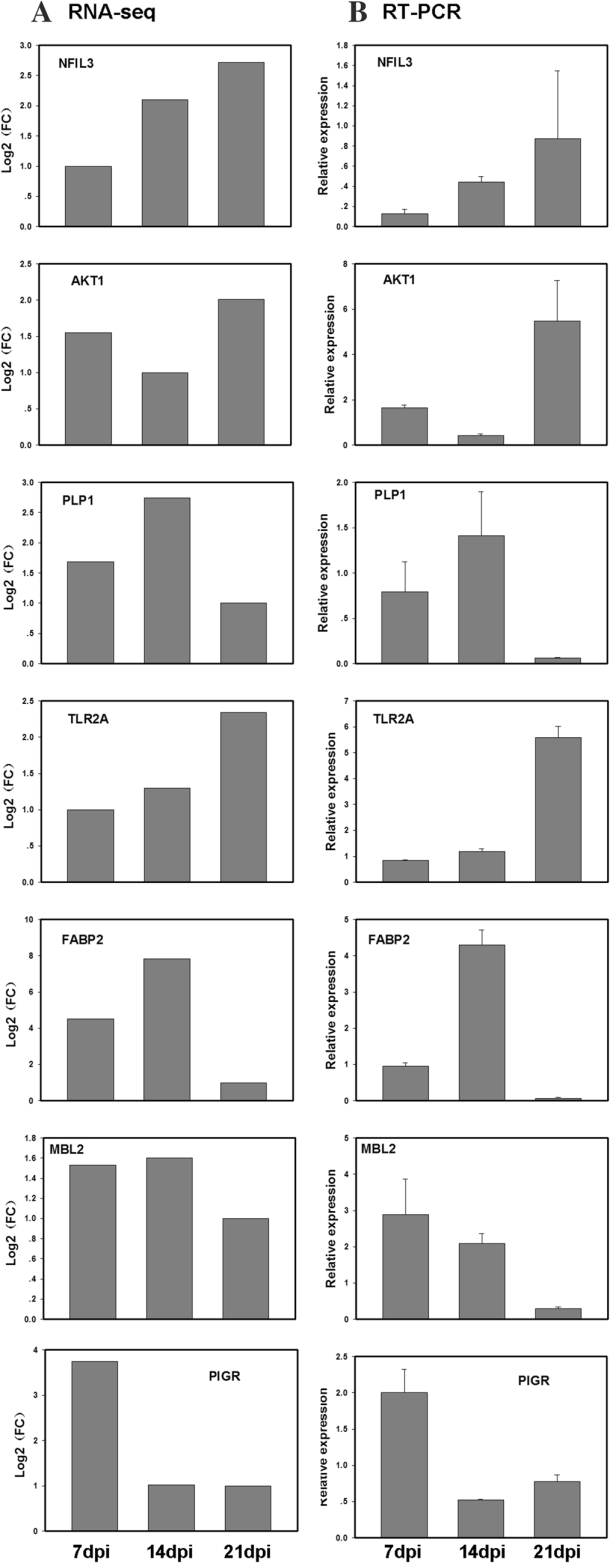
Comparison of expression levels of seven genes obtained by RNA-seq analysis (**a**) and by RT-PCR (**b**)

To validate the expression profiles of DEGs by RNA sequencing, seven DEGs were randomly selected as target genes for RT-PCR analysis. The changed patterns of the expressions for these genes at different time points obtained by RT-PCR agreed well with the values obtained by RNA-seq, although the exact fold changes slightly differed. The general expression patterns were matched indicating the reliability of the DEG results (Fig. 7).

## Discussion

FAdV-4 has been identified in several countries in association with IBH and HPS [8, 31]. The course of the disease studied under natural conditions or following experimental infection showed that the virus FAdV-4 had a high affinity towards hepatic, endothelial and lymphatic cells [13]. The most predominant and consistent gross lesions were discolored, swollen, reticulated and friable livers [32]. In the present study, livers from FAdV-4-infected chickens were swollen and yellow brown with necrotic foci (Fig. 1a). Previous studies had described intranuclear inclusion bodies in hepatocytes of naturally and experimentally infected birds [32, 33]. We also identified these intranuclear inclusion bodies (Fig. 1b). The liver is a primary FAdV-4 target and is a multifunctional organ playing important roles in metabolism, hormone production, and immunoregulation. Therefore, we chose to study the mechanisms of FAdV-4 pathogenesis and host-FAdV-4 interaction in liver tissues.

Toll-like receptors (TLRs) are type I transmembrane proteins that recognize specific signatures of invading microbes and activate a cascade of downstream signals inducing the secretion of inflammatory cytokines, chemokines, and type I interferons [34]. TLR2 and TLR9 are involved in mammalian adenovirus-induced immune responses in mice [35, 36]. In the present study, TLR1, TLR2, TLR4 and TLR7 were differentially regulated during FAdV-4 infection. Other studies had also implicated the TLRs TLR2A, TLR3 and TLR5 [17]. These results suggested that these TLRs play roles in FAdV-4-induced innate immune responses. However, the function and mechanism of these TLRs in FAdV-4 infection need further study.

The induction of type I IFN expression is an innate antiviral immune reaction in virus-infected cells [37]. In the present study, IFN expression patterns were correlated with different stages of disease progression (Table 1). For instance, the degeneration stage included suppressed IFN-α expression at 7 dpi that led to extensive viral replication and increased pathogenesis. This has been seen in other types of viral infections including the 1918 influenza virus [38], hepatitis C virus [39] and Ebola [40]. At 14 and 21 dpi, IFN-α expression showed a biphasic pattern that was up- and then downregulated that involved the convalescence and stable stages of the disease. Type II IFNs were represented only by IFN-γ that is produced by activated T cells and NK cells. This cytokine is essential for host defense against a variety of pathogens [37]. Interestingly, we found the highest IFN-γ expression at 21 dpi. The results suggested that the antiviral effect of IFNs were important in the FAdV-4-induced innate immune response.

Cytokines are a family of secreted proteins involved in immunoregulatory and inflammatory processes. IL-18 plays an important role in innate and adaptive immunity and enhances the Th1 and Th2 immune responses [41]. We identified IL-18 in our transcriptome profiles as well as in several immune signaling pathways. These results were similar to a previous study which reported that FAdV-4 infection stimulated higher mRNA expression of IL12B, IL18, CCL20 and CXCL14 in chicken liver [17]. FAdV-8 infection stimulated higher mRNA expression of IL18, IL10, and IFN-γ in chicken spleens and liver [18]. CCL19 and CCL20 are chemokines belonging to the CC chemokine family which were detected in our transcriptome profiles. CCL19 plays roles in normal lymphocyte recirculation and homing as well as in trafficking of T cells to the thymus and in T and B cell migration to secondary lymphoid organs [42, 43].

The Notch signaling pathway is an evolutionarily conserved, intercellular signaling mechanism essential for proper embryonic development in all metazoan organisms. It plays important roles in the development and function of hematopoietic stem cells, macrophages, dendritic cells, mast cells, T and B cells. It is also involved in the genesis of immune-related diseases including cancer, inflammation and autoimmune diseases [44–47]. Notch/RBP-J signaling regulates δ–γ T cell generation and migration, α–β T cell maturation, terminal differentiation of CD4(+) T cells into Th1/Th2 cells and T cell activation [48]. In the present study, the Notch signaling pathway was involved in the FAdV-4-induced immune response. Recombination signal binding protein for immunoglobulin kappa J like (RBP-JL) expression was upregulated at 7 dpi. The primary transcription factor downstream of Notch (HES, hairy and enhancer of split), was upregulated at 14 dpi. Previous studies have indicated that HES induces strong transactivation of TGF-ssRII by binding the TGF-β RII promoter through its DNA-binding domain [49]. The TGF-β signaling pathway was also involved in the FAdV-4 induced response at all three time points (Table 1). The specific regulatory mechanism should be further investigated.

In summary, the data presented in this study identified DEGs in the livers of FAdV-4 infected chickens using in vivo infection model rather than in vitro infection. We identified DEGs involved in a variety of immune-related pathways including PPAR and Notch signaling, cytokine–cytokine receptor interactions and Toll-like receptor signaling pathways. Our system analysis established a new resource for the molecular understanding of the mechanism of virus pathogenesis and may further help address how the hosts respond to FAdV-4 infection.

## Electronic supplementary material

Below is the link to the electronic supplementary material.

Supplementary material 1 (DOC 31 kb)

Supplementary material 2 (DOCX 20 kb)

Supplementary material 3 (DOCX 20 kb)

Supplementary material 4 (DOCX 18 kb)
