# Supplementary material for: Transcriptome analysis of fowl adenovirus serotype 4 infection in chickens
Source: Virus Genes. 2019 Jul 1;55(5):619–29. doi: 10.1007/s11262-019-01676-w (PMC6746880; doi:10.1007/s11262-019-01676-w)
Supplement: Supplementary file 1 — Supplementary material 1 (DOC 31 kb) [file 11262_2019_1676_MOESM1_ESM.doc]

Table S1. Validation of DEGs and their primer pairs for real-time PCR

| Gene name | Primers | Product length（bp） |
| --- | --- | --- |
| b-actin | F: 5' TGATATTGCTGCGCTCGTTG 3'  R: 5' ATACCTCTTTTGCTCTGGGCTT 3' | 183 |
| NFIL3 | F: 5' TACTCACATTCTCCGCCTCTCT 3'  R: 5' CCTTAGGAACCTGCTGCTCAT 3' | 127 |
| AKT1 | F: 5' TTACAGAACTCACGGCATCCA 3'  R: 5' CGGTCTTCAGAAAATACACGCT 3' | 143 |
| PLP1 | F: 5' TGGGTCTGTTGGAGTGCTGT 3'  R: 5' AGAGGAAGAAGAAGGAGGCTGT 3' | 243 |
| TLR2A | F: 5' TCCTGGTGGTCGTTGGGTA 3'  R: 5' CACCCAGTTGGAGTCGTTCT 3' | 155 |
| FABP2 | F: 5' TGGGCGTGAATGTGATGAA 3'  R: 5' TCGATGGTACGGAAGTTGCT 3' | 115 |
| MBL2 | F: 5' CTTCAGCCTTTCAGTGCCTTAC 3'  R: 5' CACTGAATGATGGGACAGGAAT 3' | 110 |
| PIGR | F: 5' TACGGAGCAAACGACAACG 3'  R: 5' CTTCTTTGTCTCAGCGGTGC 3' | 111 |
