# Supplementary material for: Transcriptome analysis of fowl adenovirus serotype 4 infection in chickens
Source: Virus Genes. 2019 Jul 1;55(5):619–29. doi: 10.1007/s11262-019-01676-w (PMC6746880; doi:10.1007/s11262-019-01676-w)
Supplement: Supplementary file 2 — Supplementary material 2 (DOCX 20 kb) [file 11262_2019_1676_MOESM2_ESM.docx]

Table S2. Statistical table of base information before and after filtering.

|  | Before Filtering |  |  | After Filtering |  |  |
| --- | --- | --- | --- | --- | --- | --- |
| Sample≠ | Clean Data(bp) | Q 20(%) | Q 30(%) | HQ Clean Data(bp) | Q 20(%) | Q 30(%) |
| NC-7d-1 | 10992385500 | 10718336559 (97.51%) | 10305358473 (93.75%) | 10553532547 | 10395208752 (98.50%) | 10065410476 (95.37%) |
| NC-7d-2 | 8660501700 | 8382111601 (96.79%) | 7997459864 (92.34%) | 8213753213 | 8061783043 (98.15%) | 7761357136 (94.49%) |
| NC-7d-3 | 6685867800 | 6480149076 (96.92%) | 6190260610 (92.59%) | 6362173902 | 6247061980 (98.19%) | 6018003828 (94.59%) |
| FAV-7d-1 | 8391753000 | 8197356238 (97.68%) | 7897396319 (94.11%) | 8107602181 | 7990148502 (98.55%) | 7747073036 (95.55%) |
| FAV-7d-2 | 7995576900 | 7793559670 (97.47%) | 7488614980 (93.66%) | 7695724199 | 7576551052 (98.45%) | 7333207834 (95.29%) |
| FAV-7d-3 | 7700204700 | 7519459805 (97.65%) | 7241158346 (94.04%) | 7433685621 | 7325292442 (98.54%) | 7100506453 (95.52%) |
| NC-14d-1 | 8107812000 | 7924411793 (97.74%) | 7640279572 (94.23%) | 7849172721 | 7736503924 (98.56%) | 7505184460 (95.62%) |
| NC-14d-2 | 7032033300 | 6884227395 (97.90%) | 6652578643 (94.60%) | 6821273613 | 6730296388 (98.67%) | 6540782822 (95.89%) |
| NC-14d-3 | 6778131600 | 6624306877 (97.73%) | 6386353968 (94.22%) | 6553930981 | 6460548284 (98.58%) | 6267367804 (95.63%) |
| FAV-14d-1 | 7385446200 | 7209701883 (97.62%) | 6940566387 (93.98%) | 7128249078 | 7022939320 (98.52%) | 6806153415 (95.48%) |
| FAV-14d-2 | 7157832300 | 6998521196 (97.77%) | 6750994661 (94.32%) | 6929213280 | 6831368936 (98.59%) | 6629740975 (95.68%) |
| FAV-14d-3 | 6057418200 | 5924996690 (97.81%) | 5718652170 (94.41%) | 5865130219 | 5784205684 (98.62%) | 5616077600 (95.75%) |
| NC-21d-1 | 6441880500 | 6292858186 (97.69%) | 6062766183 (94.11%) | 6225784431 | 6135888633 (98.56%) | 5949805867 (95.57%) |
| NC-21d-2 | 7286724900 | 7116127010 (97.66%) | 6852736355 (94.04%) | 7036038450 | 6933362857 (98.54%) | 6720317656 (95.51%) |
| NC-21d-3 | 6928085100 | 6757921327 (97.54%) | 6499302821 (93.81%) | 6680658266 | 6579148535 (98.48%) | 6371736188 (95.38%) |
| FAV-21d-1 | 6589533600 | 6428247551 (97.55%) | 6184131781 (93.85%) | 6348570365 | 6253176973 (98.50%) | 6058015576 (95.42%) |
| FAV-21d-2 | 8872269000 | 8660073273 (97.61%) | 8335947546 (93.96%) | 8561466038 | 8434793268 (98.52%) | 8174220610 (95.48%) |
| FAV-21d-3 | 9229546800 | 8949593686 (96.97%) | 8559138453 (92.74%) | 8791364387 | 8638221330 (98.26%) | 8341308500 (94.88%) |

Note: NC, Control, uninfected chickens; FAV, chickens Infected with FAdV-4.
