# Supplementary material for: Transcriptome analysis of fowl adenovirus serotype 4 infection in chickens
Source: Virus Genes. 2019 Jul 1;55(5):619–29. doi: 10.1007/s11262-019-01676-w (PMC6746880; doi:10.1007/s11262-019-01676-w)
Supplement: Supplementary file 3 — Supplementary material 3 (DOCX 20 kb) [file 11262_2019_1676_MOESM3_ESM.docx]

Table S3. Reads filter information and mapping reads to ribosome RNA (rRNA) database.

| Sample ≠ | Clean Reads Number | HQ Clean Reads (%) | Adapter (%) | Low quality (%) | Mapped Reads | Unmapped  Reads |
| --- | --- | --- | --- | --- | --- | --- |
| NC-7d-1 | 73282570 | 71583284 (97.68%) | 414630 (0.57%) | 1280704 (1.75%) | 1870246 ( 2.61% ) | 69713038 ( 97.39% ) |
| NC-7d-2 | 57736678 | 56014190 (97.02%) | 476854 (0.83%) | 1240038 (2.15%) | 2292168 ( 4.09% ) | 53722022 ( 95.91% ) |
| NC-7d-3 | 44572452 | 43327594 (97.21%) | 348310 (0.78%) | 892368 (2%) | 1165998 ( 2.69% ) | 42161596 ( 97.31% ) |
| FAV-7d-1 | 55945020 | 54953160 (98.23%) | 239768 (0.43%) | 752086 (1.34%) | 1427760 ( 2.60% ) | 53525400 ( 97.40% ) |
| FAV-7d-2 | 53303846 | 52215872 (97.96%) | 234466 (0.44%) | 853496 (1.6%) | 1232412 ( 2.36% ) | 50983460 ( 97.64% ) |
| FAV-7d-3 | 51334698 | 50387844 (98.16%) | 234134 (0.46%) | 712706 (1.39%) | 1477700 ( 2.93% ) | 48910144 ( 97.07% ) |
| NC-14d-1 | 54052080 | 53120286 (98.28%) | 216524 (0.4%) | 715258 (1.32%) | 1244708 ( 2.34% ) | 51875578 ( 97.66% ) |
| NC-14d-2 | 46880222 | 46134324 (98.41%) | 190024 (0.41%) | 555864 (1.19%) | 1133514 ( 2.46% ) | 45000810 ( 97.54% ) |
| NC-14d-3 | 45187544 | 44391832 (98.24%) | 201838 (0.45%) | 593868 (1.31%) | 1122232 ( 2.53% ) | 43269600 ( 97.47% ) |
| FAV-14d-1 | 49236308 | 48329026 (98.16%) | 199914 (0.41%) | 707358 (1.44%) | 1020620 ( 2.11% ) | 47308406 ( 97.89% ) |
| FAV-14d-2 | 47718882 | 46911636 (98.31%) | 190870 (0.4%) | 616370 (1.29%) | 1084240 ( 2.31% ) | 45827396 ( 97.69% ) |
| FAV-14d-3 | 40382788 | 39711494 (98.34%) | 163018 (0.4%) | 508276 (1.26%) | 803892 ( 2.02% ) | 38907602 ( 97.98% ) |
| NC-21d-1 | 42945870 | 42178194 (98.21%) | 174022 (0.41%) | 593646 (1.38%) | 1069062 ( 2.53% ) | 41109132 ( 97.47% ) |
| NC-21d-2 | 48578166 | 47685708 (98.16%) | 220670 (0.45%) | 671780 (1.38%) | 2246800 ( 4.71% ) | 45438908 ( 95.29% ) |
| NC-21d-3 | 46187234 | 45297770 (98.07%) | 176070 (0.38%) | 713388 (1.54%) | 1292726 ( 2.85% ) | 44005044 ( 97.15% ) |
| FAV-21d-1 | 43930224 | 43054430 (98.01%) | 201736 (0.46%) | 674056 (1.53%) | 1166420 ( 2.71% ) | 41888010 ( 97.29% ) |
| FAV-21d-2 | 59148460 | 58023596 (98.1%) | 254286 (0.43%) | 870568 (1.47%) | 1718998 ( 2.96% ) | 56304598 ( 97.04% ) |
| FAV-21d-3 | 61530312 | 59811620 (97.21%) | 239882 (0.39%) | 1478802 (2.4%) | 1562558 ( 2.61% ) | 58249062 ( 97.39% ) |

Note: HQ, high quality; NC, Control, uninfected chickens; FAV, Chickens Infected with FAdV-4.
