# Supplementary material for: Transcriptome analysis of fowl adenovirus serotype 4 infection in chickens
Source: Virus Genes. 2019 Jul 1;55(5):619–29. doi: 10.1007/s11262-019-01676-w (PMC6746880; doi:10.1007/s11262-019-01676-w)
Supplement: Supplementary file 4 — Supplementary material 4 (DOCX 18 kb) [file 11262_2019_1676_MOESM4_ESM.docx]

Table S4. The rRNA unmapped reads of each sample were mapped to *Gallus gallus* genome.

| Sample | Total Reads | Unmapped Reads | Unique Mapped Reads | Multiple Mapped reads | Mapping Ratio |
| --- | --- | --- | --- | --- | --- |
| NC-7d-1 | 69713038 | 9479152 (13.60%) | 58848208 (84.41%) | 1385678 (1.99%) | 86.40% |
| NC-7d-2 | 53722022 | 6741185 (12.55%) | 45963581 (85.56%) | 1017256 (1.89%) | 87.45% |
| NC-7d-3 | 42161596 | 4592510 (10.89%) | 36743108 (87.15%) | 825978 (1.96%) | 89.11% |
| FAV-7d-1 | 53525400 | 7200909 (13.45%) | 45363653 (84.75%) | 960838 (1.80%) | 86.55% |
| FAV-7d-2 | 50983460 | 7090562 (13.91%) | 42886512 (84.12%) | 1006386 (1.97%) | 86.09% |
| FAV-7d-3 | 48910144 | 6738591 (13.78%) | 41253535 (84.35%) | 918018 (1.88%) | 86.22% |
| NC-14d-1 | 51875578 | 6495429 (12.52%) | 44566833 (85.91%) | 813316 (1.57%) | 87.48% |
| NC-14d-2 | 45000810 | 5321817 (11.83%) | 38797109 (86.21%) | 881884 (1.96%) | 88.17% |
| NC-14d-3 | 43269600 | 5691986 (13.15%) | 36792404 (85.03%) | 785210 (1.81%) | 86.85% |
| FAV-14d-1 | 47308406 | 5933154 (12.54%) | 40522692 (85.66%) | 852560 (1.80%) | 87.46% |
| FAV-14d-2 | 45827396 | 5459197 (11.91%) | 39539813 (86.28%) | 828386 (1.81%) | 88.09% |
| FAV-14d-3 | 38907602 | 4817740 (12.38%) | 33403862 (85.85%) | 686000 (1.76%) | 87.62% |
| NC-21d-1 | 41109132 | 5008558 (12.18%) | 35377072 (86.06%) | 723502 (1.76%) | 87.82% |
| NC-21d-2 | 45438908 | 5698747 (12.54%) | 38881001 (85.57%) | 859160 (1.89%) | 87.46% |
| NC-21d-3 | 44005044 | 5490762 (12.48%) | 37661264 (85.58%) | 853018 (1.94%) | 87.52% |
| FAV-21d-1 | 41888010 | 5964531 (14.24%) | 35279879 (84.22%) | 643600 (1.54%) | 85.76% |
| FAV-21d-2 | 56304598 | 7726505 (13.72%) | 47608039 (84.55%) | 970054 (1.72%) | 86.28% |
| FAV-21d-3 | 58249062 | 7882398 (13.53%) | 49366922 (84.75%) | 999742 (1.72%) | 86.47% |

Note: NC, Control, uninfected chickens; FAV, Chickens Infected with FAdV-4.

Unmapped Reads, the rRNA unmapped reads; Mapping Ratio, rRNA unmapped reads were mapped to *Gallus gallus* genome.
